# Extracellular Lactate Acts as a Metabolic Checkpoint and Shapes Monocyte Function Time Dependently

**DOI:** 10.3389/fimmu.2021.729209

**Published:** 2021-11-24

**Authors:** Judith Schenz, Lena Heilig, Tim Lohse, Lucas Tichy, Katharina Bomans, Michael Büttner, Markus A. Weigand, Florian Uhle

**Affiliations:** ^1^ Department of Anesthesiology, Heidelberg University Hospital, Heidelberg, Germany; ^2^ Metabolomics Core Technology Platform (MCTP) at the Centre for Organismal Studies (COS), Heidelberg University, Heidelberg, Germany

**Keywords:** critically ill, glycolysis, immunometabolism, polyol pathway, sepsis, sorbitol, immune dysfunction

## Abstract

Elevated blood lactate levels are frequently found in critically ill patients and thought to result from tissue hypoperfusion and cellular oxygen shortage. Considering the close relationship between immune cell function and intracellular metabolism, lactate is more than a glycolytic waste molecule but able to regulate the immune response. Our aim was to elucidate the temporal and mechanistic effect of extracellular lactate on monocytes. To this end, primary human monocytes and the human monocytic cell line MonoMac6 were stimulated with various toll-like-receptor agonists after priming with Na-L-lactate under constant pH conditions. As readout, cytokine production was measured, real-time assessment of intracellular energy pathways was performed, and intracellular metabolite concentrations were determined. Irrespective of the immunogenic stimulus, short-term Na-lactate-priming strongly reduced cytokine production capacity. Lactate and hexoses accumulated intracellularly and, together with a decreased glycolytic flux, indicate a lactate-triggered impairment of glycolysis. To counteract intracellular hyperglycemia, glucose is shunted into the branching polyol pathway, leading to sorbitol accumulation. In contrast, long-term priming with Na-L-lactate induced cellular adaption and abolished the suppressive effect. This lactate tolerance is characterized by a decreased cellular respiration due to a reduced complex-I activity. Our results indicate that exogenous lactate shapes monocyte function by altering the intracellular energy metabolism and acts as a metabolic checkpoint of monocyte activation.

## Introduction

Blood lactate concentration measurement is performed routinely during care of critically ill patients. As an end product of glycolysis, its systemic occurrence is considered to be a result of cellular oxygen shortage due to tissue hypoperfusion (1). As a diagnostic criterion for septic shock, the serum lactate level has been included in the latest consensus definitions for sepsis (2) and is used to guide hemodynamic therapy (3). In addition, decreasing blood lactate concentrations are associated with a better outcome in various critical illnesses, regardless of the initial value (4).

In the field of oncology, it has been known for many years that lactic acid is not only a glycolytic waste product but an active metabolite that determines the fate of tumor cells and is able to functionally shape the immune responses within the tumor microenvironment (5). The growing evidence of the close relationship between intracellular metabolism and immune cell function (6) has ultimately led to gathered interest in lactate in the context of inflammatory diseases, too. Apart from its spatially resolved effects on innate immune cells in defined compartments (7–9), systemic effects dampening the immune response in inflammatory diseases have been described (10). Both the acidifying effect due to proton-coupled export of intracellularly produced lactate into the cell’s environment (11–14) as well as unphysiologically high lactate concentrations (15) interfere with the pro-inflammatory functions of innate immune cells. However, also concentrations measurable during illness or after intensive training have been proven to modulate the pro-inflammatory cytokine response of peripheral mononuclear cells and monocytes *in vitro* and *in vivo* (16). Monocarboxylate transporter (MCT) and the lactate dehydrogenase (LDH) activity are mechanistically involved in this dampened pro-inflammatory cytokine response. This suggests an effect independent of the already known mechanism dependent on GPR81-receptor signaling (10, 17).

Considering these results with the close relationship between blood lactate kinetics and outcome in critically ill patients, a far-reaching effect of lactate on the innate immune response, not limited to the site of origin, can be assumed. In the framework of this non-compartmentalized lactate effect, the acidotic effect can at best play a rather subordinate role. Using primary human monocytes and a monocytic cell line we aimed to further elucidate the MCT-LDH axis mediated effect of extracellular lactate on the cytokine response under physiological pH, focusing on its temporal and mechanistical resolution.

## Materials and Methods

### Cell Lines and Reagents

**Figure 1:**
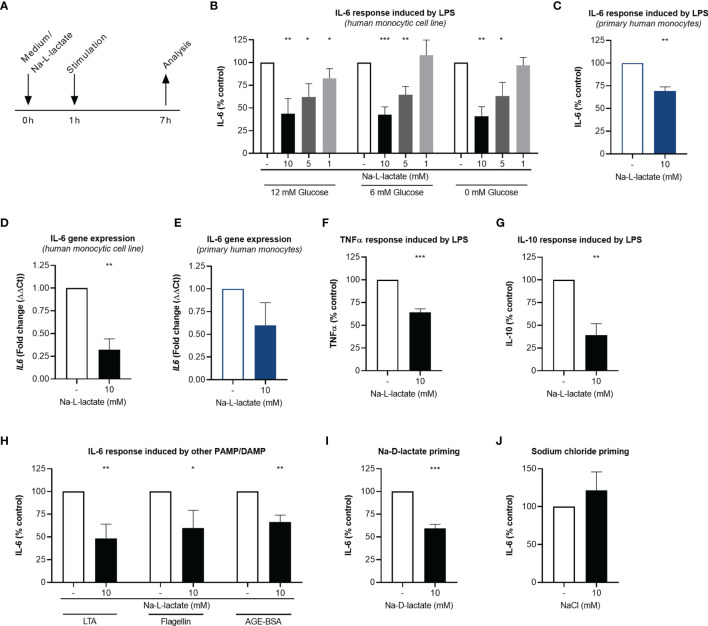
Extracellular lactate reduces cytokine response upon stimulation. **(A)** Time scale of the analyses. **(B)** IL-6 secretion from MM6 primed with Na-L-lactate at the indicated concentrations for one hour and stimulated with LPS for six hours in medium with 12mM, 6mM, or without glucose. **(C)** IL-6 secretion from primary human monocytes primed with 10mM Na-L-lactate for one hour and stimulated with LPS for six hours. Gene expression of *IL6* in MM6 **(D)** and primary human monocytes **(E)** after one-hour priming with 10mM Na-L-lactate and six hours stimulation with LPS. **(F)** TNFα and **(G)** IL-10 secretion from MM6 primed with 10mM Na-L-lactate for one hour and stimulated with LPS for six hours. **(H)** IL-6 secretion from MM6 after one-hour priming with 10mM Na-L-lactate and stimulation with LTA, flagellin, or AGE-BSA for six hours. IL-6 secretion from MM6 primed with 10mM **(I)** Na-D-lactate or **(J)** NaCl for one hour and stimulated with LPS for six hours. Data are shown as mean + SD (n=4; except for **(C)** and **(E)** n=3). One sample t test was performed against the theoretical value 100 or 1 respectively (***P ≤ 0.001, **P ≤ 0.01, *P ≤ 0.05).

The human monocytic cell line MonoMac6 (MM6) which was purchased from DSMZ-German Collection of Microorganisms and Cell Cultures GmbH (Braunschweig, Germany). Cells were cultivated using RPMI1640 medium containing GlutaMAX supplemented with 1mM sodium pyruvate, MEM Non-Essential Amino Acids Solution (all from Thermo Fisher Scientific Inc., Waltham, USA), and 10% heat inactivated fetal bovine serum ultra-low endotoxin (Cell Concepts, Umkirch, Germany). If not stated otherwise, cells were used at a density of 5x10^5^ cells/mL and experiments were performed using RPMI1640 medium without glucose supplemented with MEM Non-Essential Amino Acids Solution (both from Thermo Fisher Scientific Inc., Waltham, USA) and 10% heat inactivated fetal bovine serum ultra-low endotoxin (Cell Concepts, Umkirch, Germany). Unless stated otherwise, D(+)-Glucose (Merck KGaA, Darmstadt, Germany) was added with a final concentration of 6mM. Sodium L-lactate (Na-L-lactate), sodium D-lactate (Na-D-lactate) [both ≥99.0% (NT)], and sodium chloride (NaCl) (all from Merck KGaA, Darmstadt, Germany) were used for priming at the indicated concentrations. The LDH inhibitor GSK 2837808A (CAS Number 1445879-21-9) (Tocris Bioscience, Bristol, United Kingdom) was dissolved in dimethyl sulfoxide (DMSO) and used at a final concentration of 10µM. Stimulation was performed with 100ng/mL ultrapure lipopolysaccharide (LPS) (from *Escherichia coli*, strain O111:B4), 10µg/mL purified lipoteichoic acid (LTA) (from *Staphylococcus aureus*), 100ng/mL ultrapure flagellin (from *Pseudomonas aeruginosa*) (all from Invivogen, San Diego, USA), or 100µg/mL Glycoaldehyde-Advance Glycation End Products modified BSA (AGE-BSA) (BioVision, Milpitas, USA). In general, cells were primed for one hour with Na-L-lactate and then stimulated with LPS for six hours before subsequent analyses (**Figure 1A**).

### Isolation of Primary Human Monocytes

Untouched primary human monocytes were isolated by magnetic-activated cell sorting (MACS) using the Pan Monocyte Isolation Kit (Miltenyi Biotec, Bergisch Gladbach, Germany). Peripheral whole blood of healthy volunteers anticoagulated with lithium heparin was diluted 2:1 with phosphate-buffered saline (PBS) (Thermo Fisher Scientific Inc., Waltham, USA), transferred to Leucosep tubes prefilled with separation medium for PBMC isolation (greiner bio-one, Kremsmuenster, Austria), and centrifuged (800xg, 15min, no brake). After density gradient centrifugation, the peripheral blood mononuclear cells (PBMC) layer was aspirated carefully and washed three times (250xg, 5min, 4°C) with ice-cold isolation buffer (PBS supplemented with 2mM ethylenediaminetetraacetic acid (Thermo Fisher Scientific Inc, Waltham, Mass) and 0.5% protease-free bovine serum albumin (Carl Roth, Karlsruhe, Germany)). For indirect magnetic labeling, each 10^7^ PBMC were first incubated with 40µl of isolation buffer, 10µl of FcR Blocking Reagent, and 10µl of Biotin-Antibody Cocktail for 5min at 4°C. After adding of additional 30µl of isolation buffer and 20µl of Anti-Biotin Micro Beads per 10^7^ cells the mixture was incubated at 4°C for another 10min. Separation was performed using an AutoMACS™ Pro Separator (Miltenyi Biotec, Bergisch Gladbach, Germany) and the program “Depletes”. The negative fraction (unlabeled cells) containing the isolated monocytes was collected. The purity of isolated cells was verified by flow cytometry (data not shown).

Cells were used at a density of 5x10^5^ cells/mL and experiments were performed using RPMI1640 medium containing GlutaMAX (glucose concentration: 12mM) supplemented with 10% heat inactivated fetal bovine serum ultra-low endotoxin (Cell Concepts, Umkirch, Germany).

### Cytokine Analysis

For cytokine analysis, supernatant was collected and stored at -20°C until further analysis. Cytokine levels were determined using colorimetric enzyme-linked immunosorbent assay (ELISA) (Human IL-6 and Human TNF-alpha DuoSet ELISA and Human IL-10 Quantikine ELISA; all from R&D Systems, Minneapolis, USA) as indicated by the manufacture. An Epoch™ 2 microplate spectrophotometer (BioTek, Instruments GmbH, Bad Friedrichshall, Germany) and the accompanying software Gen5™ was used for all absorbance measurements.

### Quantitative Polymerase Chain Reaction

Ribonucleic acids (RNA) were isolated using a commercially available column-based approach (RNeasy Plus Mini Kit, Qiagen, Hilden, Germany) according to the manufacture’s instruction including β-mercaptoethanol (Carl Roth, Karlsruhe, Germany) addition to the lysis buffer. Concentrations were determined measuring the absorbance at 230nm spectrophotometrically (Nanodrop, Thermo Fisher Scientific, Waltham, USA) and 500ng RNA were used for reverse transcription using the Quantitect Reverse Transcription Kit (Qiagen, Hilden, Germany) and Mastercycler nexus X2 (Eppendorf, Hamburg, Germany). Quantitative polymerase chain reaction was performed on a StepOnePlus Real-Time PCR System using commercially available reagents (TaqMan Fast Advanced Master Mix) and TaqMan assays (assay IDs: HK2: Hs00606086_m1, HCAR1: Hs02597779_s1, IL6: Hs00174131_m1, LDHA: Hs01378790_g1, LDHB: Hs00929956_m1, PKM: Hs00987255_m1, SLC2A1: Hs00892681_m1, SLC2A4: Hs00168966_m1; SLC5A12: Hs01054645_m1, SLC16A1: Hs01560299_m1, SLC16A3: Hs00358829_m1, SLC16A7: Hs00940851_m1, SLC16A8: Hs00895133_g1, ACTB: Hs01060665_g1) (all Thermo Fisher Scientific Inc., Waltham, USA). The reactions were carried out in triplicates. Gene expression was calculated as fold change compared to control. In detail, the values of the gene of interest were subtracted from the respective values of the endogenous control gene (ACTB) yielding ΔCt, followed by calculation of ΔΔCt=2^(ΔCt_treated_–ΔCt_untreated_).

### pH Measurements

Extracellular medium pH was assessed using a pH meter (pH 211 Microprocessor pH Meter with pH electrode HI1131B; both Hanna instruments, Vöhringen, Germany) and calibration solutions (Buffer solution pH 4.0 and pH 7.0; both Honeywell, Morristown, USA).

Intracellular acidification was measured using pHrodo Green AM Intracellular pH Indicator (Thermo Fisher Scientific Inc., Waltham, USA) as indicated by the manufacture. Briefly, pHrodo dye was added 1:10 into PowerLoad concentrate and this mixture then was further diluted 1:100 in Live Cell Imaging Solution (140mM NaCl, 2.5mM potassium chloride, 1.8mM calcium chloride, 1mM magnesium chloride (all from Merck KGaA, Darmstadt, Germany), and 20mM HEPES ((Carl Roth, Karlsruhe, Germany); pH 7.4). Growth medium was replaced by this staining solution and cells were incubated at 37°C for 30 minutes (min) prior to measurement. This was done either directly using cells from culture or after one-hour priming with Na-L-lactate and six hours stimulation with LPS. As a positive control, the respiratory chain was inhibited by antimycin A and rotenone (both Agilent, Santa Clara, USA). A FACSVerse cytometer (BD Biosciences, Franklin Lakes, USA) was used for measurement. Results were calculated using mean fluorescent intensity.

### Live Cell Metabolic Analysis

**Figure 2:**
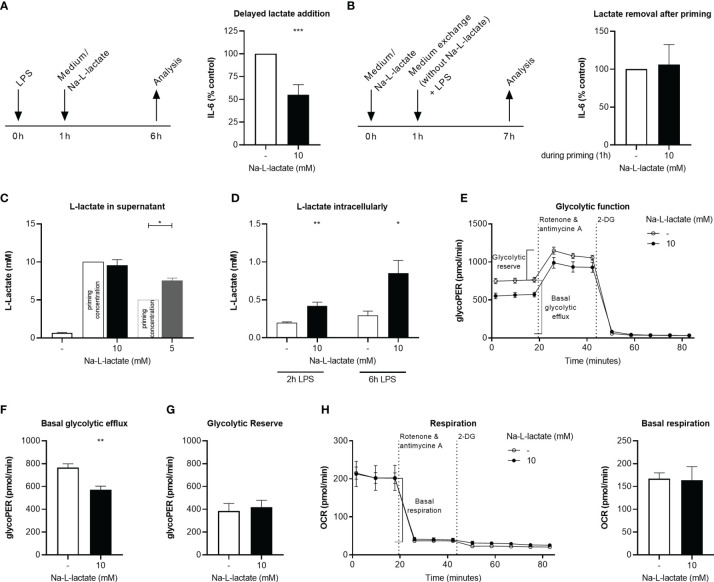
Intracellular lactate accumulation hinders glycolytic flux. **(A)** IL-6 secretion from MM6 after six hours stimulation with LPS and addition of Na-L-lactate on hour after the start of stimulation. **(B)** IL-6 secretion after one-hour priming with 10mM Na-L-lactate which was removed prior to a six-hour stimulation with LPS. L-lactate concentration in **(C)** the supernatant or **(D)** intracellularly after one-hour priming with Na-L-lactate at the indicated concentration and two- or six-hours stimulation with LPS. **(E)** Calculation of the glycolytic indices **(F)** basal glycolytic efflux and **(G)** glycolytic reserve after one-hour priming with 10mM Na-L-lactate and six hours stimulation with LPS. **(H)** Calculation of basal respiration one-hour priming with 10mM Na-L-lactate and six hours stimulation with LPS. Data are shown as **(A, B)** mean + SD or **(C, H)** mean + SEM (n=5; except for **(C)** n=3). Group comparisons were performed by unpaired t test, except for **(A, C),** where one sample t tests were performed against the theoretical value 100, 10, or 5 respectively (***P ≤ 0.001, **P ≤ 0.01, *P ≤ 0.05).

Glycolytic proton efflux rate (glycoPER) and oxygen consumption rate (OCR) were determined by Seahorse technology (Agilent Technologies, Santa Clara, USA). Cells were primed and stimulated as indicated. Afterwards the cells were harvested, counted, and seeded (7x10^4^ cells/well) into Seahorse XFp Cell Culture Miniplates (Agilent Technologies, Santa Clara, USA) previously coated with Cell-Tak (Corning, Corning, USA). The plate was centrifuged (300x*g*, 1min, no brake) and incubated in a non-CO_2_ 37°C incubator for 30min. Seahorse XF Base Medium (without Phenol Red) supplemented with 5mM HEPES (both from Agilent Technologies, Santa Clara, USA), 10mM D(+)-Glucose (Merck KGaA, Darmstadt, Germany), and 2mM L-glutamine (Thermo Fisher Scientific Inc., Waltham, USA) was used as assay medium. Following three baseline measurements (each measuring point comprises 1.5min mixing, 3min waiting, and 3min measuring) 1µM rotenone/antimycin A (Seahorse XFp Glycolytic Rate Assay Kit, Agilent, Santa Clara, USA), and 50mM 2-Deoxy-D-glucose (2-DG) (Merck KGaA; Darmstadt, Germany) were added sequentially. Wave 2.6.0 software was used for analysis and parameters were calculated using Seahorse XF Glycolytic Rate Assay Report Generator 3.21 (**Figure 2E**) and manually based on the equations from Seahorse XF Cell Mito Stress Test Report Generator 3.0.11 (**Figure 2F**) (all three from Agilent Technologies, Santa Clara, USA).

### L-Lactate Measurement

For intracellular L-lactate assessment, 5x10^6^ cells were primed and stimulated as indicated. Thereafter, cells were harvested, washed twice with PBS (Thermo Fisher Scientific Inc., Waltham, USA), and resuspended in 100µL PBS and 50µL 0.6N HCl (Honeywell, Morristown, USA). For proper cell disruption, samples were sonicated [3 cycles each consisting of 30 seconds (sec) power (on) followed 30sec rest (off)] using Bioruptor Pico (Diagenode, Liège, Belgium). Sample pH was neutralized by adding 50µL 1M Tris base (Carl Roth, Karlsruhe, Germany) and supernatant was collected after centrifugation (14,000x*g*, 15min). For extracellular L-lactate determination, supernatant was collected. All sample preparation steps were performed at 4°C and samples were stored at -80°C until analysis. L-lactate concentrations were measured using the commercially available Amplite Colorimetric L-Lactate Assay Kit (AAT Bioquest, Sunnyvale, USA) following the manufacture’s instruction.

### NAD^+^/NADH Measurement

5x10^5^ cells were primed and stimulated as indicated. Subsequently, cells were pelleted and resuspended in 200µL cold PBS (Thermo Fisher Scientific Inc., Waltham, USA) and 200µL cold base solution (100mM sodium carbonate, 20mM sodium bicarbonate, 0.05% Triton X-100, 10mM nicotinamide, and 1% dodecyltrimethylammonium bromide [all from Merck KGaA, Darmstadt, Germany)] and stored at -80°C until further analysis. Directly before measurement, each 50µL sample were heated at 60°C for 15min either together with 25µL 0.4N HCl (Honeywell, Morristown, USA) or alone to destroy the NADH or NAD^+^ respectively. The acidified sample was neutralized with 25µL 0.5M Tris base (Carl Roth, Karlsruhe, Germany). To adjust volume and composition 25µL HCl and 25µL 0.5M Tris base were added to the other sample as well. NAD^+^ and NADH levels were assessed using the NAD/NADH-Glo Assay (Promega, Mannheim, Germany) as indicated by the manufacture. Measurement was performed on a LUMIstar^®^ (BMG Labtech, Ortenberg, Germany) with a gain of 4095.

### Aldo-Keto Reductase Enzyme Activity Measurement

1x10^6^ cells were primed with L-lactate for one hour and stimulation with LPS for six hours. Subsequently, cells were harvested, resuspended in 200µL assay buffer included in the Aldo-Keto Reductase Activity Assay Kit (Colorimetric) (abcam, Cambridge, United Kingdom), and incubated on ice for 30min. After centrifugation (13,000x*g*, 10min, 4°C) supernatant was collected and stored at -80°C. Aldo-keto reductase activity was determined following the manufactures’ instructions.

### Measurement of Mitochondrial Superoxide Production

Mitochondrial superoxide production was measured after one-hour priming with Na-L-lactate and three hours stimulation with LPS using MitoSOX™ Red reagent (Thermo Fisher Scientific Inc., Waltham, USA) as indicated by the manufacture. To prepare a staining solution, the stock solution was diluted 1:1000 with Hank’s balanced salt solution (HBSS) (Thermo Fisher, Scientific Inc., Waltham, USA). Cells were harvested, washed once with pre-warmed HBSS and staining solution was added. After incubation at 37°C for 10min, cells were washed again with pre-warmed HBSS before measurement. A FACSLyric cytometer (BD Biosciences, Franklin Lakes, USA) was used for measurement. Results were calculated using mean fluorescent intensity. Cells in which the respiratory chain was inhibited by antimycin A and rotenone (both Agilent, Santa Clara, USA) were used as a positive control. Complex I enzyme activity measurement 2x10^6^ cells were primed with L-lactate for 24 hours. Subsequent sample preparation from pelleted cells and complex I enzyme activity measurement was performed using Complex I Enzyme Activity Assay Kit (Colorimetric) (abcam, Cambridge, United Kingdom) as indicated by the manufacture. Sample protein concentration was determined using the Pierce™ BCA™ Protein-Assay (Thermo Fisher Scientific Inc., Waltham, USA) and samples were diluted to a concentration of 700µg protein/mL.

### Metabolite Analysis by Gas Chromatography/Mass Spectrometry

5x10^6^ cells were primed with L-lactate for one hour and stimulated with LPS for six hours. Thereafter, cells were harvested, washed twice with 0.9% NaCl (Merck KGaA, Darmstadt, Germany), pellets were snap frozen in liquid nitrogen, and stored at -80°C until extraction. Extraction was done in 360µL of 100% methanol for 15min at 70°C with vigorous shaking. As internal standard, 20µL ribitol (0.2mg/mL) was added to each sample. After the addition of 200µL chloroform samples were shaken at 37°C for 5min. To separate polar and organic phases, 400µL water were added and samples were centrifuged for 10min at 11,000x*g*. For the derivatization, 700µL of the polar (upper) phase were transferred to a fresh tube and dried in a speed-vac (vacuum concentrator) without heating.

Pellets were re-dissolved in 20µL methoximation reagent containing 20mg/mL methoxyamine hydrochloride (Sigma 226904) in pyridine (Sigma 270970) and incubated for two hours at 37°C with shaking. For silylation, 32.2µL N-Methyl-N-(trimethylsilyl)trifluoroacetamide (MSTFA; Sigma M7891) and 2.8µL Alkane Standard Mixture (50mg/mL C10 - C40; Fluka 68281) were added to each sample. After incubation for 30min at 37°C, samples were transferred to glass vials for GC/MS analysis.

A GC/MS-QP2010 Plus (Shimadzu^®^) fitted with a Zebron ZB 5MS column (Phenomenex^®^; 30meter x 0.25mm x 0.25µm) was used for GC/MS analysis. The GC was operated with an injection temperature of 230°C and 1µL sample was injected with split mode (1:10). The GC temperature program started with a 1min hold at 40°C followed by a 6°C/min ramp to 210°C, a 20°C/min ramp to 330°C and a bake-out for 5min at 330°C using helium as carrier gas with constant linear velocity. The MS was operated with ion source and interface temperatures of 250°C, a solvent cut time of 7min and a scan range (m/z) of 40–700 with an event time of 0.2sec. The “GCMS solution” software (Shimadzu^®^) was used for data processing.

### Statistical Analysis

All statistical analyses were done in GraphPad Prism (V 8.0.2 for Windows, GraphPad software, La Jolla, USA). Results are visualized as mean + standard error of the mean (SEM) in case of absolute numbers or mean + standard deviation (SD) for normalized data. Normal distribution was tested by Shapiro-Wilk normality test. Statistical comparison between two groups was done using the unpaired t test in case of normal distributed data. For group comparisons between more than two groups displaying normal distribution, one-way ANOVA was performed followed by Fisher’s LSD test. In case of normalized data, one sample t test against the theoretical value resulting from normalization (usually, 1 respectively 100) was performed. Statistical significance was considered with *P* ≤ 0.05.

## Results

### Stimulation Induced Cytokine Production Is Reduced After Extracellular Lactate Priming

To investigate the impact of extracellular lactate on the inflammatory response, MM6 cells were primed with increasing concentrations of Na-L-lactate and subsequently stimulated with the Toll-like receptor (TLR) 4 agonist LPS for six hours (**Figure 1A**). IL-6 production was diminished in a dose dependent manner (**Figure 1B**). As standard RPMI1640 media formulation contains 12mM glucose, we performed the same experiment using RPMI1640 medium containing 6mM glucose, thereby mimicking a physiological, normoglycemic environment. A comparable pattern of reduced IL-6 production after lactate priming was found (**Figure 1B**). Surprisingly, performing the experiments in medium without glucose addition revealed similar results, indicating that extracellular glucose concentration is not an influencing factor. Therefore, normoglycemic medium with 6mM glucose was used for all further experiments. The same effect of extracellular lactate on cytokine response was also observed in freshly isolated primary human monocytes from healthy donors (**Figure 1C**). 24-hour stimulation led to a comparable decrease in cytokine release from MM6 cells and primary human monocytes (**Supplementary Figure 1**). After lactate priming, the expression of the *IL6* gene was strongly reduced after LPS stimulation compared to unprimed cells in the cell line (**Figure 1D**) as well as in primary cells (**Figure 1E**), hinting towards a transcriptional regulation induced by extracellular lactate. In addition, we found the effect not to be restricted to IL-6, since TNFα (**Figure 1F**) and IL-10 (**Figure 1G**) production were also affected. Stimulating the cells with either the TLR 2 agonist LTA, the TLR 5 agonist flagellin, or AGE-BSA resulted in significant lower IL-6 production after lactate priming, too (**Figure 1H**). Moreover, priming with the enantiomer Na-D-lactate also reduces the IL-6 production in response to LPS stimulation (**Figure 1I**). To exclude the sodium ions being responsible for the observed effect, we primed the cells with isomolar concentrations of NaCl and no changes in cytokine production were found (**Figure 1J**). In summary, priming with extracellular lactate leads to a diminished global cytokine production in response to various pathogen- and damage-associated molecular patterns *via* a reduced expression of the corresponding gene.

Na-L-lactate was used to avoid pH changes and associated effects. Nevertheless, we controlled medium pH to ensure proper culture conditions. Compared to control cells, medium pH was stable one hour after priming (**Supplementary Figure 2A**) as well as six hours after stimulation (**Supplementary Figure 2B**). Going beyond, priming with extracellular lactate might be thought to influence intracellular pH despite extracellular stability leading to the described altered cytokine production. Addressing this, we stained the cells with an intracellular pH indicator (**Supplementary Figure 2C**). Compared to control cells, no difference could be reported in lactate primed cells neither rapidly (0.5 hours) after priming (**Supplementary Figure 2D**) nor after subsequent stimulation with LPS (6 hours) (**Supplementary Figure 2E**). These results prove that the observed effects can be attributed to the lactate at physiological pH and are not an acidification artefact.

### Extracellular Lactate Leads to Intracellular Lactate Accumulation Resulting in Decreased Glycolytic Flux

Administering extracellular lactate one hour after the immunogenic stimulus led to the same result of a reduced IL-6 production (**Figure 2A**), indicating that exact timing of lactate administration is not decisive. In contrast, removing the extracellular lactate after priming prior to stimulation fully abolished the effect (**Figure 2B**). It might therefore be assumed that the extracellular lactate needs to be taken up by the cells to induce intracellular changes ultimately leading to the altered cytokine response. However, six hours after addition of 10mM lactate, an amount of 9.6 +/- 0.7mM L-lactate was detectable in the supernatant (**Figure 2C**). When lower amounts (5mM) were added to the supernatant, the cells were still able to export net additional lactate, which led to a significant increase in extracellular L-lactate concentration (7.5 +/- 0.3mM). Unprimed cells secreted 0.6 +/- 0.1mM L-lactate upon stimulation. These results suggest that there is a certain threshold concentration for extracellular lactate in the range of 10mM up to which the cell is capable of exporting net lactate. Despite stable extracellular lactate levels when primed with 10mM, intracellular L-lactate levels were significantly higher than in control cells six hours after stimulation with LPS (0.9 +/- 0.2mM vs. 0.3 +/- 0.1mM; *P*=0.0079) (**Figure 2D**). Interestingly, intracellular accumulation is a fast process: Although delayed lactate administration induces the phenotype, too, already two hours after stimulation there is a significant difference between cells primed with extracellular lactate and not-primed cells. These let to the conclusion, that extracellular lactate alters intracellular lactate flux. Glycolytic flux measurement using Seahorse technology (**Figure 2E**) further substantiated this. Basal glycolytic efflux after priming with extracellular lactate and stimulation with LPS was lowered (**Figure 2F**) while glycolytic reserve was not affected (**Figure 2G**). These changes do not result from an altered expression of transporters involved in either lactate or glucose handling since no changes in the expression of genes coding for MCT-1, MCT-2, MCT-4, and Glut1 transporter were found in response to lactate priming (**Supplementary Figure 3**). Other transporters known for their role in cellular lactate (GPR81, SMCT2, or MCT-3) or glucose handling (Glut4) are not expressed in MM6 cells (data not shown). The glycolytic changes were not accompanied by changes of cellular respiration (**Figure 2H**). Taken together, extracellular lactate leads to a decreased glycolytic flux as a result of intracellular lactate accumulation.

### Lactate Priming Reduces NAD^+^ Availability *via* Glycolytic Shutdown

**Figure 3:**
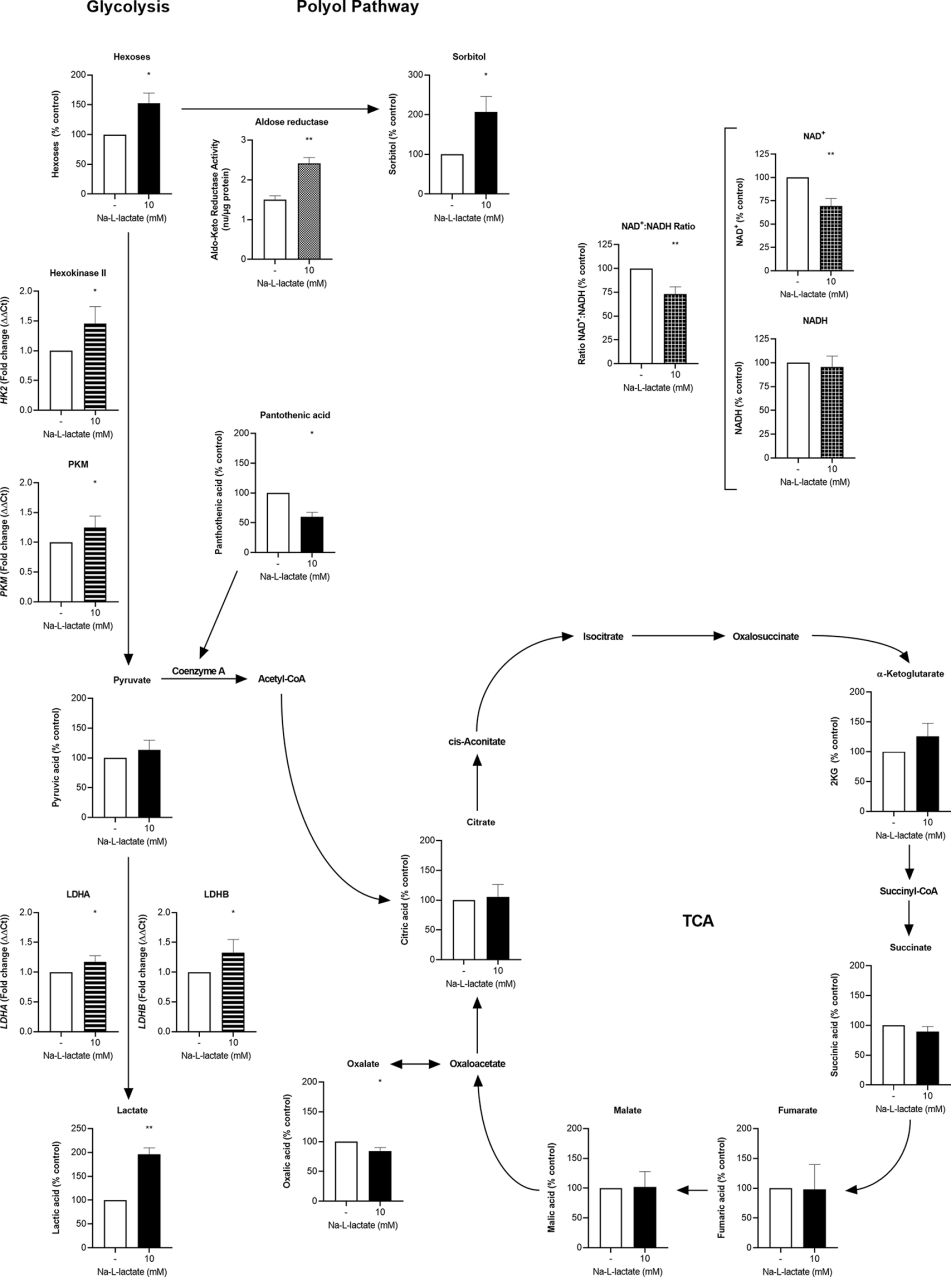
Extracellular lactate plugs glycolysis and increases turnover in the branching polyol pathway. Intracellular concentration (filled columns) of hexoses, sorbitol, pyruvate, lactate, pantothenic acid, citrate, α-ketoglutarate, succinate, fumarate, malate, oxalate (n=3), NAD^+^, NADH (white checkered columns), and aldose reductase (white dotted column) activity, and gene expression (white striped columns) of *HK2*, *PKM*, *LDHA*, and *LDHB* (n=5) after one-hour priming with 10mM Na-L-lactate and six hours stimulation with LPS. Data are shown as mean + SD, except for aldose reductase activity, where mean + SEM are shown. One sample t tests were performed against the theoretical values 100 or 1 respectively. Group comparison for aldose reductase activity was performed by unpaired t test (**P ≤ 0.01, *P ≤ 0.05).

The inhibition of LDH with an inhibitor (GSK 2837808A) also let to a lower IL-6 response to LPS stimulation (**Supplementary Figure 4A**). Cells primed with lactate displayed a lower intracellular NAD^+^-to-NADH ratio, with decreased levels of NAD^+^ and preserved NADH levels (**Figure 3**). Comparable to intracellular lactate accumulation, these changes were already observable shortly after stimulation for 2h (**Supplementary Figure 4B**). Since no differences in oxygen consumption were found (**Figure 2F**), comparable NAD^+^ usage in mitochondrial TCA cycle can be assumed, indicating that the hindrance of the LDH reaction and decreased NAD^+^ regeneration is underlying the NAD^+^ reduction. Using mass spectrometry, we were able to confirm the intracellular lactate accumulation, while we did not find alterations in the levels of neither pyruvate nor the TCA cycle intermediates citrate, α-ketoglutarate, succinate, fumarate, and malate. Oxalate and pantothenic acid levels were diminished. Of importance, we detected an accumulation of hexoses and sorbitol. This further proves the shutdown of glycolysis and, together with the increased aldose reductase activity, indicates a shunt of glucose into the branching polyol pathway, with this member of the aldo-keto reductase family being the pathway’s rate limiting enzyme. Concomitant, the gene expression of hexokinase II, the enzyme initiating glycolysis and previously being described as lactate-influenced (18), was slightly increased as well as the rather downstream glycolytic enzymes PKM, LDH-A, and LDH-B. Using primary human monocytes, we observed similar changes in gene expression (**Supplementary Figure 5A**) and a slightly reduced production of mitochondrial superoxide (**Supplementary Figure 5B**). Altogether, these findings reveal extracellular lactate acting as a “glycolysis plug”.

### Long-Term Priming Induces Cellular Adaption Abrogating the Suppressive Effect

**Figure 4:**
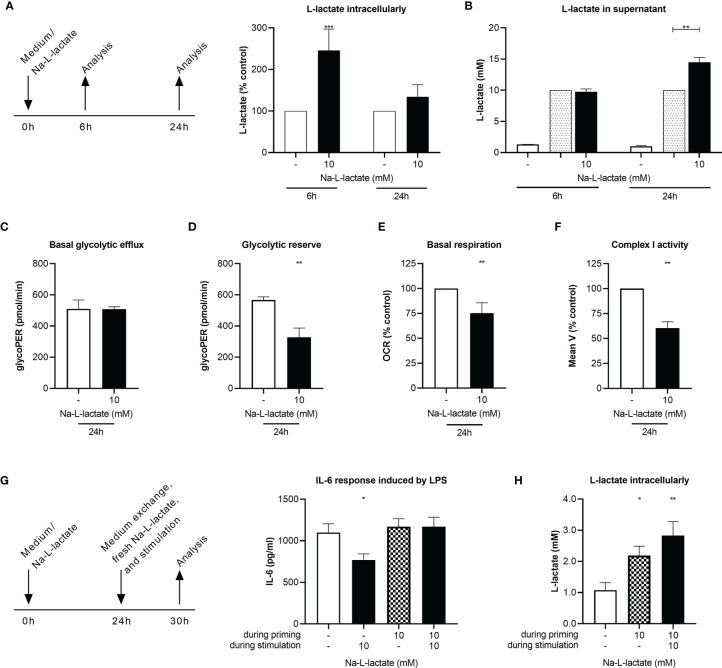
Long-term priming induces lactate resistance. **(A)** Intracellular and **(B)** supernatant L-lactate concentration after a six- or 24-hour priming with 10mM Na-L-lactate. **(C)** Basal glycolytic efflux, **(D)** glycolytic reserve, **(E)** basal respiration, and **(F)** activity of complex I of the respiratory chain after a 24-hour priming with 10mM Na-L-lactate. **(G)** IL-6 secretion and **(H)** intracellular L-lactate concentration after 24 hours priming with Na-L-lactate as indicated and subsequently stimulation for six hours with LPS in the presence of fresh 10mM Na-L-lactate as indicated. Data are shown as **(A, E, F)** mean + SD or **(B, D, G, I)** mean + SEM (n=5; except for **(B)** n=4 and **(F)** n=3). Group comparisons were performed by **(C, D)** unpaired t test or **(G, H)** one-way ANOVA followed by Fisher’s LSD test. **(A, B, E, F)** One sample t tests were performed against the theoretical values 100 or 10 respectively (***P ≤ 0.001, **P ≤ 0.01, *P ≤ 0.05).

While administering lactate close to stimulation resulted in a blockage of glycolysis, we next asked how the cells react when they are exposed for a prolonged time to lactate without an additional stimulation pressure. We found a massive intracellular lactate accumulation after priming the cells for six hours just with lactate (**Figure 4A**), accompanied by slightly lower NAD^+^ levels and again preserved NADH levels (**Supplementary Figure 6**). Interestingly, this effect was abolished with further extension of the incubation time. After 24 hours, intracellular lactate was only slightly increased compared to control cells. The reason for this seems to be cellular adaptions which enable the cells to release intracellularly accumulated lactate, as illustrated by the increased concentrations in the supernatant after 24h of priming (14.5 +/- 0.8mM), going beyond the previously added amount (10mM) (**Figure 4B**). After only six hours priming, the extracellular concentration was still unaltered. A cellular adaptation must have taken place, which is proven by stable glycolytic efflux rates after 24 hours lactate priming (**Figure 4C** and **Supplementary Figure 7A**) and reduced glycolytic reserve (**Figure 4D** and **Supplementary Figure 7A**). Mitochondrial adjustment can be observed as a consequence of long-term lactate priming: Basal respiration was lowered (**Figure 4E** and **Supplementary Figure 7B**) due to a reduced activity of the respiratory chain’s complex I (**Figure 4F**). These adaptions are leading to a “lactate tolerance”: when cells are stimulated with LPS after 24-hour lactate priming in the presence of freshly added lactate, no reduction of IL-6 production is observed (**Figure 4G**). Interestingly, lactate-primed cells still show an additional accumulation of intracellular lactate compared to completely medium-primed cells, regardless of whether extracellular lactate was added during stimulation or not (**Figure 4H**). Taken together, long-term priming with extracellular lactate induces cellular adaption that leads to an abrogation of the suppressive effect.

## Discussion

We here provide evidence for extracellular lactate acting as a metabolic checkpoint and shaping monocyte function provoking Janus-faced phenotypes depending on the exposure time. Short-term priming, with regard to the inflammatory stimulus, leads to a global reduction of cytokine production, whereas long-term exposure induces cellular adaption going along with lactate tolerance. The early effect might serve as a negative feedback loop to counteract excessive acute inflammatory reactions and prevent collateral harm to the body. In healthy volunteers, a comparable effect has already been proven: Artificially increased blood lactate levels due to breathing exercises were involved in protection against subsequent LPS-induced inflammatory response (19). In contrast, a long-term suppressive effect on the innate immune response could lead to harmful effects, such as a higher susceptibility for secondary and opportunistic infections and viral reactivation (20), and contribute to immune dysfunction (21, 22). Considering the situation in critically ill patients with decreasing lactate levels predicting beneficial outcome (4, 23, 24) and the high monocyte turnover under inflammatory conditions (25), blood monocytes *in vivo* are rarely exposed to high blood lactate levels for a sufficiently long time to induce the observed cellular adaption prior to an immunogenic stimulation. Nevertheless, the adaption process inside the monocytes might be important in the context of a local immune response in hypoperfused tissues or compartments. To further elucidate the mechanisms of such a spatial lactate effect shaping organ-specific immunology, an *in vivo* approach is necessary. Our *in vitro* approach, which focuses on the intracellular mechanisms, cannot adequately answer this question. However, the temporally resolved different effects revealed by our mechanistic analysis also raise the question of a possible therapeutic application, which needs to be investigated in further studies. Various application possibilities are conceivable: Especially in diseases associated with acute systemic inflammatory reactions, lactate infusion therapy could have a beneficial, counter-regulatory effect, but if administered at the wrong time, could also further promote a hyporesponsive state rendering the host susceptible for nosocomial infections, as known from sepsis (26–28). Thus, exact therapy timing is required due to the Janus-phased phenotype. In addition, local treatment can be considered in hyperinflammatory disorders such as chronic inflammatory bowel disease or autoimmune diseases (7). The cellular adaptation by long-term priming and hence the abrogation of the suppressive effect could be beneficial in the context of preconditioning in preparation of circumstances that cause high blood lactate concentrations such as surgical procedures to avoid lactate-induced hyporesponsive immune states.

Unlike several other studies (11–14), our data provide evidence that lactate at physiological pH is responsible for the observed phenotypical changes and neither extra- nor intracellular acidification. On a mechanistic level, short-term priming with extracellular lactate shapes monocyte function by blocking glycolysis. A comparable effect on glycolysis has already been described in mouse macrophages (15) and in CD14^+^ monocytes from healthy donors (16). In contrast to Ratter et al., we could not detect any change in oxidative rates after short-term priming. A reason for this difference could be that the timeframe between lactate administration and analysis was significantly shorter than in our approach. Consensus, however, is that inhibition of glycolysis shifts the use of metabolic pathways within the cell. Thus, we identified a massive upregulation of the polyol pathway going along with glycolysis inhibition. As known from diabetes research (29), this induction results from intracellular hyperglycemia, which in our case is a consequence of the lactate-induced glycolysis backlog. While the latter cannot be prevented even by counter-regulatory, increased glycolytic enzyme expression, the upregulation of the polyol pathway is facilitated by a higher activity of the pathway’s rate limiting enzyme aldose reductase. A higher consumption of NADPH and secondarily lower availability as cofactor for other enzymes can be concluded. Secondly, glycolysis inhibition results in a depletion of the intracellular NAD^+^ pool mainly by hindering the LDH reaction. This interference of extracellular lactate with the cellular redox balance reshapes enzyme activities and subsequently processes related to the inflammatory response.

Poly(ADP-ribose)polymerase-1 (PARP-1) is part of a large, NAD^+^ consuming family of enzymes playing not only an important role in DNA damage repair but also catalyzing post-translational protein modifications shaping gene expression and amplifying inflammation (30). In LPS-induced acute lung injury models, PARP-1 inhibition blocked NF-κB activation (31) and was able to reduce the expression of NF-κB-dependent genes in lung tissue (32). Moreover, in a cecal ligation and puncture sepsis model, the PARP inhibitor olaparib displayed protective effects and extended survival by reducing organ injury and modulating inflammation (33). Extracellular lactate might act as an indirect PARP inhibitor *via* the reduced NAD^+^ availability resulting in the observed attenuated monocytic function. Reduced intracellular NAD^+^ concentrations also interfere directly with TLR signaling by impeding protein phosphorylation within the MyD88-dependent signaling cascade in monocytes (34). Furthermore, in a LPS-induced sepsis mouse model inhibiting the nicotinamide phosphoribosyltransferase, the key enzyme in the NAD^+^ salvage pathway which is important for glyceraldehyde 3-phosphate dehydrogenase activity and therefore glycolysis sustentation, diminished disease severity during the very early phase of the disease (35).

TLR receptor signaling induces acetyl-CoA generation *via* upregulation of glycolytic flux. This is a prerequisite for histone acetylation and subsequent pro-inflammatory gene expression (36). Lactate priming reduces this fuel source for histone acetylation not only *via* the inhibited glycolytic flux but might in addition hinder the generation of acetyl-CoA by β-oxidation through the reduced NAD^+^ availability. The diminished content of pantothenic acid, a precursor for *de novo* synthesis of CoA (37), further supports the concept of lactate induced derangements of acetyl-CoA dependent mechanisms.

Besides altering histone acetylation, Zhang et al. only recently identified lysine residues in histones as a direct target for lactate. While gene expression of pro-inflammatory cytokines was not changed, metabolic processes such as the glutathione metabolism and response to hypoxia were found to be affected in a delayed kinetic needing 16 to 24 hours after initiation of intracellular lactate accumulation (38). The induction of such a “lactate clock” might be involved in the cellular adaption leading to lactate tolerance through long-term priming with extracellular lactate. Whether the observed reductions in the glycolytic reserve and the activity of respiratory chain’s complex I result from such a mechanism, however, warrants further research. These changes can also be explained by a shifted utilization of pyruvate: To counteract the glycolytic backlog and keep the glycolytic efflux at a constant level, a higher amount of pyruvate is used to drive the LDH reaction. This is underpinned by the observed reduction in the glycolytic reserve, which means a lower increase in glycolytic efflux after inhibition of the mitochondrial respiratory chain and indicates that the glycolytic enzymes in total are not altered, but in lactate-primed cells, a higher proportion of the available enzyme activity is already utilized under basal conditions. The reduced complex I activity, in turn, may reflect a lower refueling of the TCA cycle by pyruvate, which is available in smaller amounts compared to unprimed cells, as larger amounts are used to drive the LDH reaction. Regardless of the underlying mechanism, abated activity of the respiratory chain’s complex I is known to increase inflammation and additionally lactate was shown to further augment this process (39, 40). This is in line with our findings that a lower complex I activity resulting from long-term lactate-priming nullifies the acute lactate effect.

Our results provide valuable insights, but the chosen experimental approach also implies limitations. We have been working mainly with an immortalized monocytic cell line and repeated some experiments with primary monocytes for confirmation. Since large cell numbers were required, this reduces the big effort of isolating primary monocytes from the blood of healthy donors. At the same time, this approach minimizes the risk that cellular characteristics differing from primary cells due to immortalization are causative for the results and ensures robust and reproducible results. Many aspects of critical care medicine influence the immune system, hence, studying monocytes isolated from patients with high blood lactate concentrations and therefore exposed *in vivo* is crucial to translate the findings into clinic. Similarly, animal models are required to further substantiate the evidence of the results shown here as a basis for the use of lactate therapy in the context of systemic inflammatory responses, both therapeutically and preventively.

Taken together, our results suggest that extracellular lactate acts as a metabolic checkpoint independent of the already known GPR81-signaling dependent mechanism and shapes monocyte function *via* distinct acute and long-term effects. While glycolysis is blocked in the acute setting and glucose is shunted into the branching polyol pathway instead, the long-term effect induces an adaption reaction altering the use of glycolysis derived pyruvate. Therefore, a temporary elevation of systemic lactate may be considered an evolutionary mechanism of the organism to counteract excessive inflammation.

## Data Availability Statement

The original contributions presented in the study are included in the article/**Supplementary Material**. Further inquiries can be directed to the corresponding author.

## Ethics Statement

The studies involving human participants were reviewed and approved by the ethics committee of the Medical Faculty of the Heidelberg University (S-823/2020). The participants provided their written informed consent to participate in this study.

## Author Contributions

JS, MW, and FU designed the study and planned methodology. JS, LH, TL, LT, and KB conducted the experiments. MB performed metabolite analysis by GC/MS. JS, MW, and FU performed data interpretation and statistics and wrote the manuscript. All authors critically revised and agreed to the submitted manuscript.

## Conflict of Interest

The authors declare that the research was conducted in the absence of any commercial or financial relationships that could be construed as a potential conflict of interest.

## Publisher’s Note

All claims expressed in this article are solely those of the authors and do not necessarily represent those of their affiliated organizations, or those of the publisher, the editors and the reviewers. Any product that may be evaluated in this article, or claim that may be made by its manufacturer, is not guaranteed or endorsed by the publisher.

## Abbreviations

AGE-BSA, Glycoaldehyde-Advance Glycation End Products modified BSA; DMSO, dimethyl sulfoxide; ELISA, enzyme-linked immunosorbent assay; GC, Gas Chromatography; glycoPER, Glycolytic proton efflux rate; HBSS, Hank’s balanced salt solution; LDH, lactate dehydrogenase; LPS, lipopolysaccharide; LTA, lipoteichoic acid; MCT, monocarboxylate transporter; MFI, mean fluorescence intensity; MM6, MonoMac6; MS, Mass Spectrometry; MSTFA, N-Methyl-N-(trimethylsilyl)trifluoroacetamide; OCR, oxygen consumption rate; PBS, phosphate-buffered saline; PARP-1, poly(ADP-ribose)polymerase-1; RNA, ribonucleic acids; TLR, toll-like receptor.
