# Supplementary material for: Extracellular Lactate Acts as a Metabolic Checkpoint and Shapes Monocyte Function Time Dependently
Source: Front Immunol. 2021 Nov 24;12:729209. doi: 10.3389/fimmu.2021.729209 (PMC8652120; doi:10.3389/fimmu.2021.729209)
Supplement: Supplementary file 1 [file DataSheet_1.pdf]

Supplementary Material

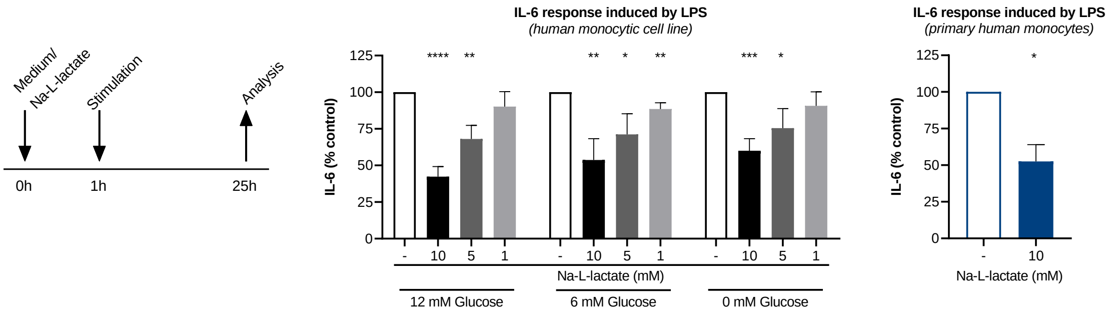

Supplementary Figure 1. **Extracellular lactate reduces cytokine response upon stimulation.** IL-6 secretion from MM6 primed with Na-L-lactate at the indicated concentrations for one hour and stimulated with LPS for 24 hours in medium with 12mM, 6mM, or without glucose (n=5) and from primary human monocytes primed with Na-L-lactate for one hour and stimulated with LPS for 24 hours in medium with 12mM glucose (n=3). Data are shown as mean + SD. One sample t test was performed against the theoretical value 100 (**** $P<0.0001$, *** $P\leq0.001$, ** $P\leq0.01$, * $P\leq0.05$).

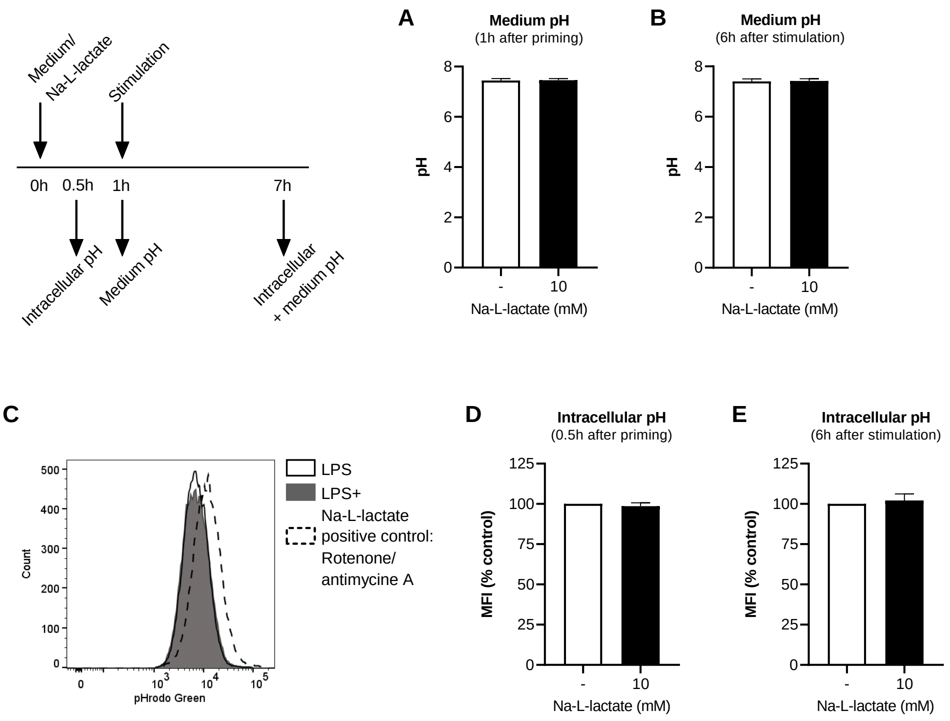

Supplementary Figure 2. **Physiological pH is maintained despite lactate priming.** Medium pH after **(A)** one-hour priming with Na-L-lactate and **(B)** additional six hours stimulation with LPS. **(C)** Intracellular pH after **(D)** 30min priming with Na-L-lactate or **(E)** one-hour priming with Na-L-lactate and six hours stimulation with LPS. Data are shown as (A, B) mean + SEM or (D, E) mean + SD (n=3).

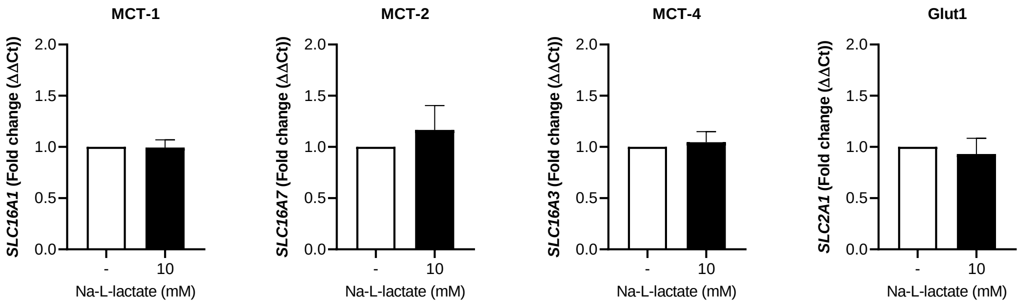

Supplementary Figure 3. **Expression levels of lactate and glucose transporter are maintained.** Gene expression of *SLC16A1*, *SLC16A7*, *SLC16A3*, and *SLC2A1* after one-hour priming with 10mM Na-L-lactate and six hours stimulation with LPS. Data are shown as mean + SD (n=5).

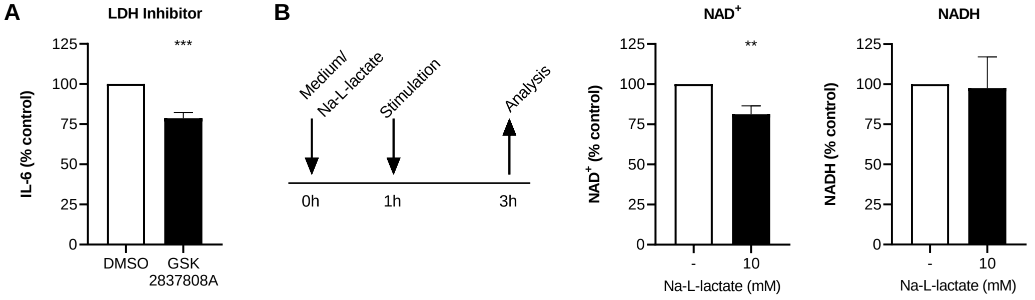

Supplementary Figure 4. **Role of LDH. (A)** IL-6 secretion after inhibition of LDH starting one hour prior to a six-hour stimulation with LPS. **(B)** Intracellular NAD^+ and NADH levels after one-hour priming with 10 mM Na-L-lactate and two hours stimulation with LPS. Data are shown as mean + SD (n=5). One sample t test was performed against the theoretical value 100 (*** $P \leq 0.001$).

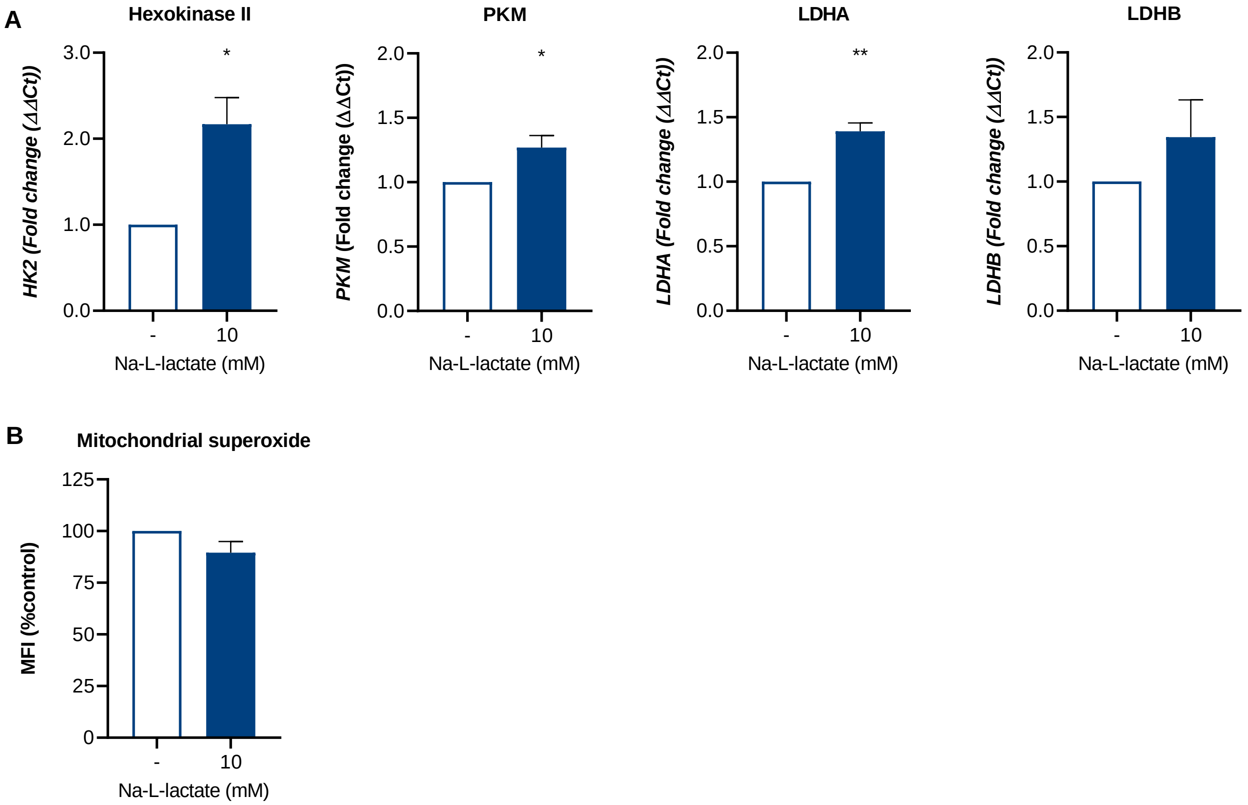

Supplementary Figure 5. **Glycolytic enzyme gene expression and mitochondrial superoxide production in primary monocytes. (A)** Gene expression of *HK2*, *PKM*, *LDHA*, and *LDHB* in primary human monocytes after one-hour priming with 10mM Na-L-lactate and six hours stimulation with LPS. **(B)** Production of mitochondrial superoxide in primary human monocytes after one-hour priming with 10mM Na-L-lactate and three hours stimulation with LPS. Data are shown as mean + SD (n=3). One sample t tests were performed against the theoretical values 100 or 1 respectively (** $P \leq 0.01$, * $P \leq 0.05$).

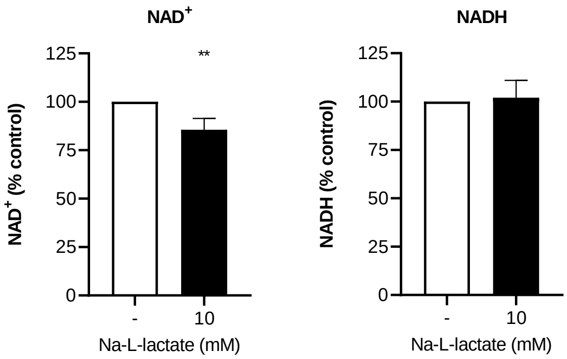

Supplementary Figure 6. **Extracellular lactate alone is sufficient to reduce NAD^+ level.** Intracellular NAD^+ and NADH level safter six hours priming with 10mM Na-L-lactate. Data are shown as mean + SD (n=5). One sample t test was performed against the theoretical value 100 (** $P \leq 0.01$).

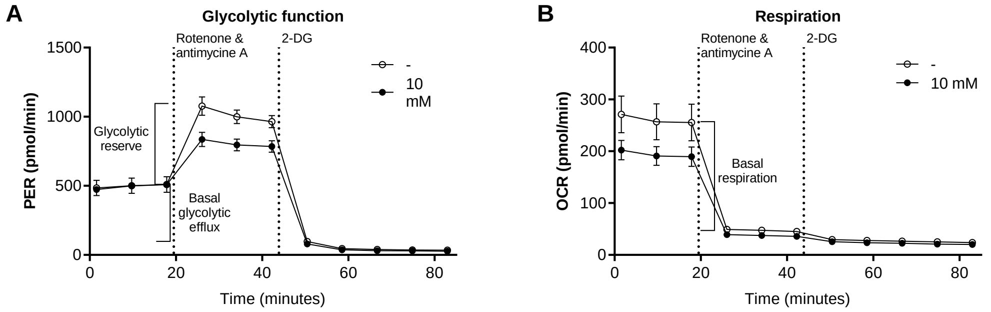

Supplementary Figure 7. **Calculation of glycolytic and respiratory indices. (A)** Calculation of the glycolytic indices basal glycolytic efflux and glycolytic reserve and **(B)** calculation of basal respiration after a 24-hour priming with 10mM Na-L-lactate. Data are shown as mean +/- SEM (n=5).
